# Long-Term Use of an App-Based Intervention for Older Adults’ Quality of Life: SMART-AGE First Insights

**DOI:** 10.1093/geroni/igaf122.2810

**Published:** 2025-12-31

**Authors:** Nicole Memmer, Anna Schlomann, Hans-Werner Wahl

**Affiliations:** Heidelberg University, Heidelberg, Baden-Wurttemberg, Germany; Network Aging Research, Heidelberg, Baden-Wurttemberg, Germany; Heidelberg University, Heidelberg, Baden-Wurttemberg, Germany

## Abstract

With growing expectations for technology to enhance the quality of life in older adults, the ongoing SMART-AGE intervention study investigates the benefits of a multi-domain, app-based intervention designed to promote (1) social participation, (2) physical fitness, and (3) health awareness across a 6-month observation period. The design is a three-armed, randomized controlled trial, including 649 community-dwelling older adults (67+ years) with basic digital skill level, assigned to a full intervention, partial intervention, and active control condition. In addition to evaluating the effectiveness of the intervention, analyzing automatically tracked app usage data is central to the study, providing insights into adherence and engagement patterns. To this end, this poster presents initial findings on the use of one of the implemented apps, i.e., smartVERNETZT, which is a German adaptation of the proven PRISM-App in the U.S. (CREATE consortium; Czaja et al,) aimed to foster social participation. Among the 286 participants analyzed for long-term usage, 111 (39%) used smartVERNETZT weekly during the final three months, meeting the criteria for long-term users. Besides income and education, no significant baseline differences in social connectedness, technology skills, or attitudes toward technology between long-term and non-long-term users of smartVERNETZT were found. Additionally, a statistically significant decline in usage was noted, with engagement dropping from an average of 2.74 days per week in the first three months to 2.15 days per week in the last three months. Findings underline the challenge of sustaining long-term engagement in digital interventions for older adults.

